# Hypoxia inducible factor (HIF) 3α prevents COPD by inhibiting alveolar epithelial cell ferroptosis via the HIF-3α-GPx4 axis

**DOI:** 10.7150/thno.99237

**Published:** 2024-08-28

**Authors:** Junchao Jiang, Zhoude Zheng, Shengsong Chen, Jixiang Liu, Ju Jia, Yuhang Huang, Qing Liu, Chung Y Cheung, Don D Sin, Ting Yang, Chen Wang

**Affiliations:** 1 China-Japan Friendship Hospital (Institute of Clinical Medical Sciences), Chinese Academy of Medical Sciences & Peking Union Medical College, Beijing, CN.; 2 National Center for Respiratory Medicine; State Key Laboratory of Respiratory Health and Multimorbidity, Beijing, CN.; 3 The University of British Columbia, Centre for Heart Lung Innovation, St. Paul's Hospital, Vancouver, BC, CA.; 4 Peking University China-Japan Friendship School of Clinical Medicine, Beijing, CN.; 5 First Affiliated Hospital of Nanchang University, Department of Pulmonary and Critical Care Medicine, Nanchang, Jiangxi, CN.; 6 Department of Infectious Disease, Beijing Friendship Hospital, Capital Medical University, Beijing, CN.; 7 The University of British Columbia, Division of Respiratory Medicine, Department of Medicine, Vancouver, BC, CA.

**Keywords:** HIF-3α, pre-COPD, hypoxia, ferroptosis, emphysema

## Abstract

**Rationale:** COPD patients are largely asymptomatic until the late stages when prognosis is generally poor. In this study, we shifted the focus to pre-COPD and smoking stages, and found enrichment of hypoxia inducible factor (HIF)-3α is in pre-COPD samples. Smoking induced regional tissue hypoxia and emphysema have been found in COPD patients. However, the mechanisms underlying hypoxia especially HIF-3α and COPD have not been investigated.

**Methods:** We performed bulk-RNA sequencing on 36 peripheral lung tissue specimens from non-smokers, smokers, pre-COPD and COPD patients, and using “Mfuzz” algorithm to analysis the dataset dynamically. GSE171541 and EpCAM co-localization analyses were used to explore HIF-3α localization. Further, *Sftpc^Creert2/+^R26^LSL-Hif3a^* knock-in mice and small molecular inhibitors *in vitro* were used to explore the involvement of HIF-3α in the pathophysiology of COPD.

**Results:** Reactive oxygen species (ROS) and hypoxia were enriched in pre-COPD samples, and HIF-3α was downregulated in alveolar epithelial cells in COPD. *In vitro* experiments using lentivirus transfection, bulk-RNA seq, and RSL3 showed that the activation of the HIF-3α-GPx4 axis inhibited alveolar epithelial cell ferroptosis when treated with cigarettes smoking extracts (CSE). Further results from *Sftpc^Creert2/+^R26^LSL-Hif3a^* knock-in mice demonstrated overexpression of HIF-3α inhibited alveolar epithelial cells ferroptosis and prevented the decline of lung function.

**Conclusion:** Hypoxia and oxidation-related damage begins years before the onset of COPD symptoms, suggesting the imbalance and impairment of intracellular homeostatic system. The activation of the HIF-3α-GPx4 axis is a promising treatment target. By leveraging this comprehensive analysis method, more potential targets could be found and enhancing our understanding of the pathogenesis.

## Introduction

Chronic obstructive pulmonary disease (COPD) is a leading cause of morbidity and mortality worldwide with most patients developing the disease over decades 1. However, patients are largely asymptomatic or minimally symptomatic until the disease progresses to advanced stages when the prognosis is generally poor 2-4. Given that there are no curative therapies for COPD, the Lancet Commission introduced 1) genetics, 2) early-life events, 3) pulmonary infections, 4) tobacco smoke exposure, 5) pollution and pre-COPD for future research and a better understanding of COPD pathogenesis. 5,6.

In this study, we conducted a comprehensive analysis by including the asymptomatic pathological stages. Based on etiology (smoking) and pathological lesions (pre-COPD), we divided the pathological process into four groups: 1) non-smokers; 2) smokers; 3) pre-COPD (which refers to individuals with structural lung lesions (e.g., emphysema) without airflow limitation (FEV_1_/FVC ≥ 0.7 post-bronchodilation)); 4) COPD, and conducted a bulk-RNA sequencing of these 36 peripheral lung specimens (Table 1).

With the declining gas exchange capacity, patients with COPD frequently suffer from hypoxia and hypoxemia 7. Previous reports have emphasized the association of hypoxia and hypoxemia with emphysema in COPD 8,9. Emphysema is characterized by the destruction of gas-exchanging surfaces of the alveolar epithelial cells. Poorly oxygenated blood from emphysema areas is mixed with properly oxygenated blood in the left heart and consequently causes lower oxygen saturation in arterial blood 10. Smokers with Sp_O2_ <92% had higher emphysema percentage values as compared with those with Sp_O2_ >92%, and a strong modifying effect of emphysema percentage on Sp_O2_ 9.

Prolonged hypoxia exposure imposes the regulation of target genes by hypoxia-inducible factors (HIFs) and causes the shift of mitochondrial oxidative phosphorylation to anaerobic glycolysis 11,12. The accumulating mitochondrial-ROS (mtROS) further aggravates the imbalance of oxidative stress, ultimately leading to cell death and a series of subsequent pathological lesions like emphysema, mucus accumulation and small airway dysfunction 11,13. Although systemic hypoxia and severe emphysema are seen in COPD, the mechanisms underlying hypoxia, especially HIF-3α and emphysema in COPD have not been investigated.

In the present study, using the omics technology on human tissue samples obtained from non-smokers, smokers, pre-COPD and COPD patients, we found significant HIF expression pattern changes in the pre-COPD stage. Furthermore, *in vitro* experiments and *Sftpc^Creert2/+^R26^LSL-Hif3a^* knock-in mice demonstrated that -activation of the HIF-3α-GPx4 axis prevents COPD by inhibiting alveolar epithelial cell ferroptosis.

## Results

### The Mfuzz algorithm reveals core clusters

**Figure 1:**
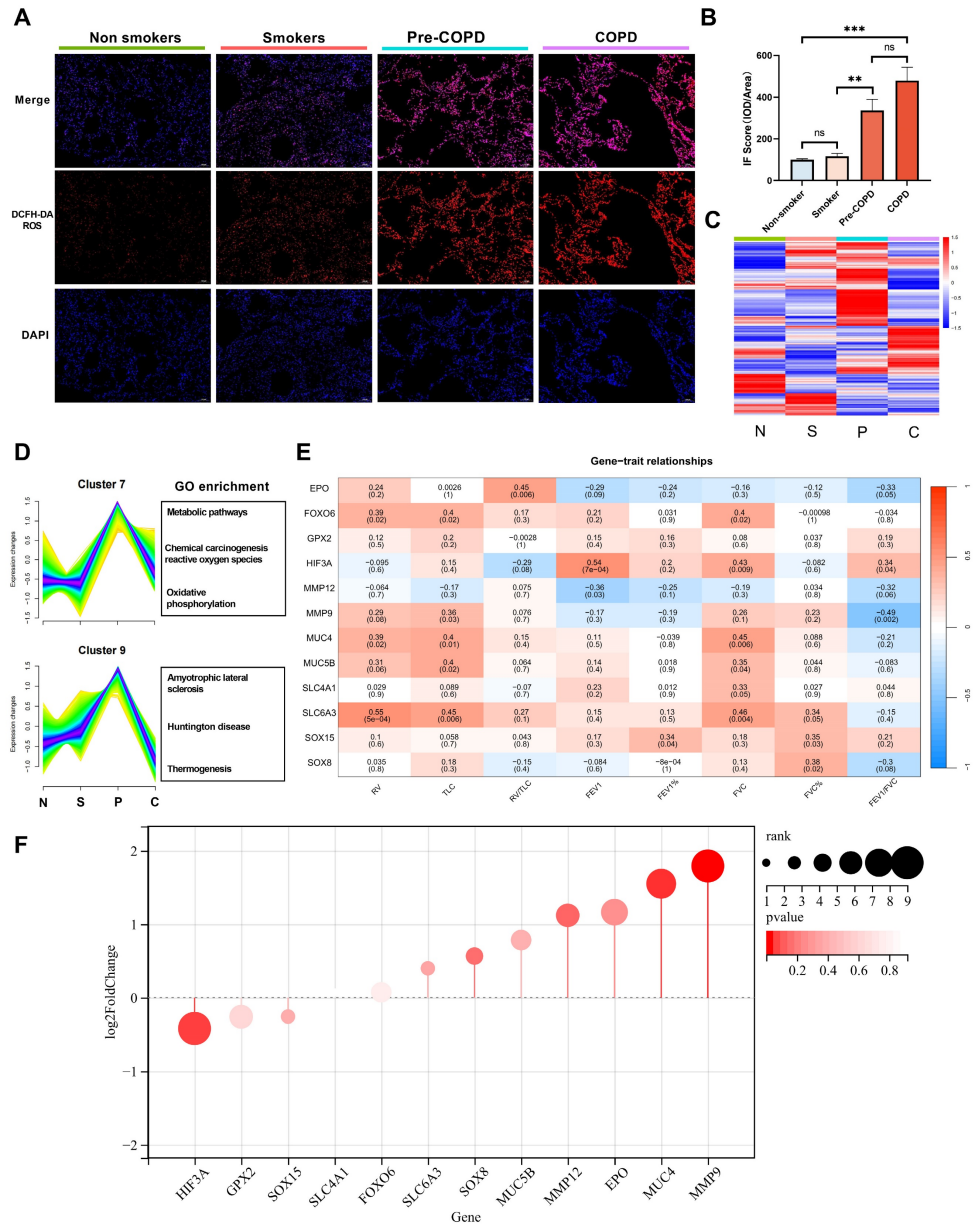
** bulk-RNA sequencing identified core gene HIF-3α. A-B)** Cellular ROS level was examined by DCFH-DA probe in each sample. Original magnification ×10. Scale bars: 100 μm. **C)** Heatmap of bulk-RNA sequencing data of the 4 groups. “N” stands for nonsmokers; “S” stands for smokers; “P” stands for pre-COPD; “C” stands for COPD. **D)** Mfuzz based pseudo-time analysis and gene ontology annotation analysis. “N” stands for nonsmokers; “S” stands for smokers; “P” stands for pre-COPD; “C” stands for COPD.** E)** Core gene and spirometry correlation analysis, color in red represent positive correlation, in blue represent negative correlation.** F)** Rank list of the core genes, the larger size represents the higher log_2_FC value, color in red represents p-value. IOD: integrated optical density. **: p < 0.01; ***: p < 0.005; ns: p > 0.05 (non-significant).

We included 36 lung specimens from non-smokers, smokers, pre-COPD and COPD patients (Table 1). Using reactive oxygen species (ROS) probe on these lung samples, we found ROS level was significantly increased in pre-COPD (Figure 1A-B) stage. We performed bulk-RNA seq to those four groups (Table 1, Figure 1C and GSE275503), and using Mfuzz algorithm to analyze this dataset dynamically. Mfuzz generated 10 clusters (Figure S1) and among them, cluster 7 and cluster 9 attracted our attention as they showed a strong peak in pre-COPD, which is consistent with the ROS character in human lung specimens (Figure 1A). By further gene ontology annotation analysis, we found cluster 7 has more redox biology related terms enriched (Figure 1D). We further extract the gene expression value (RPKM) in cluster 7 and performed a correlation analysis with lung function (Figure 1E), blood count (Figure S2) and blood biochemistry test (Figure S3). According to the results, HIF-3α is correlated with FEV_1_/FVC positively; whereas MMP9 has a negative correlation. These two genes also showed the largest log_2_-fold-change (log_2_FC) values and the most significant p values in this transcriptomic dataset (Figure 1F). Based on HIF-3α is a transcription activator that can functioned in a more widely way, we next focused on HIF-3α 14,15.

### Explore HIF-3α cellular localization

**Figure 2:**
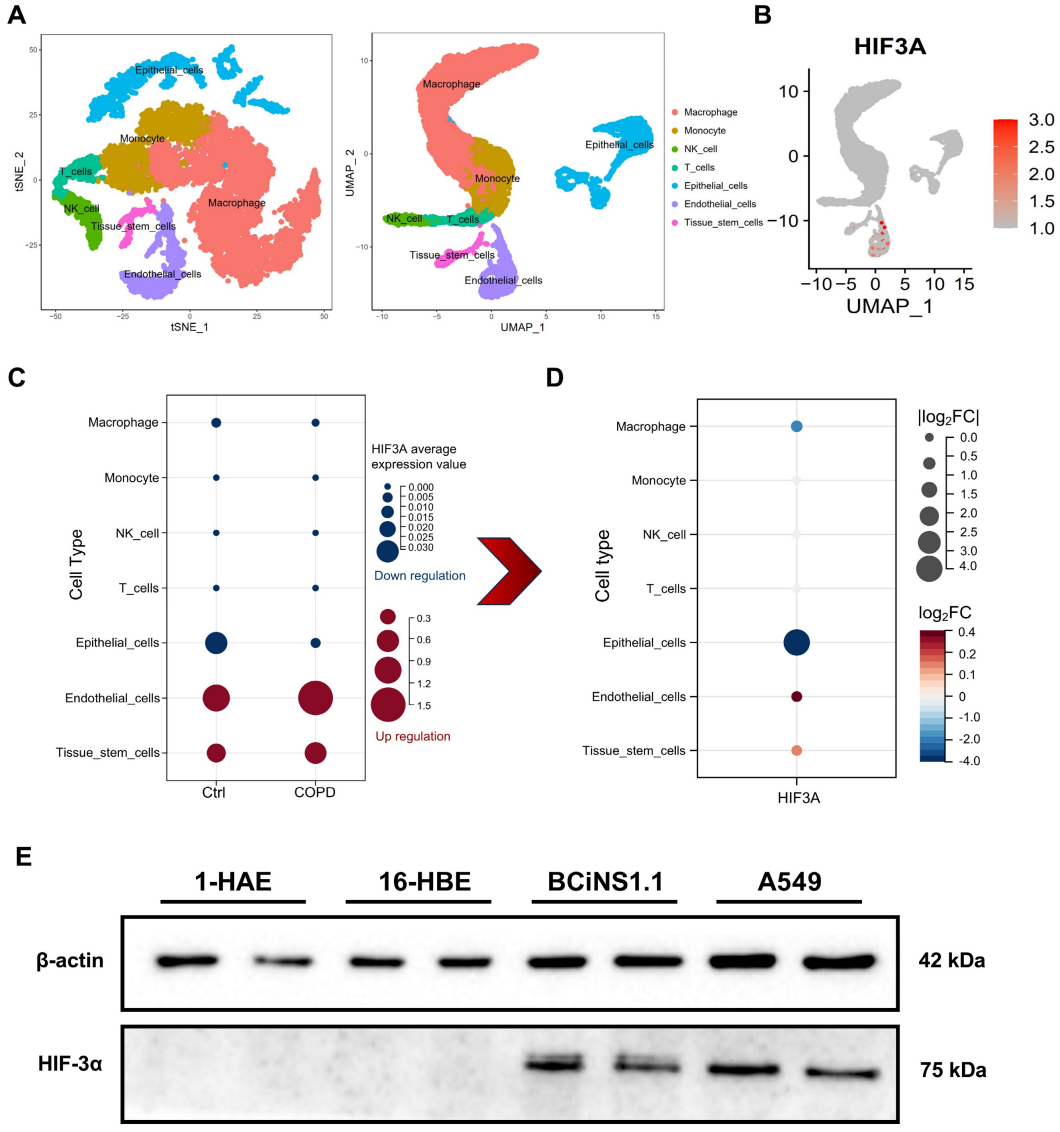
** Explore HIF-3α cellular localization. A)** t-SNE and UMAP plot shows the annotation of 7 kinds of cell types in scRNA-seq. **B)** UMAP-based expression profile of HIF-3α. **C)** In sub-group expression featuring plot of HIF-3α, color in blue represents the downregulation in COPD; in red represents the upregulation in COPD. **D)** HIF-3α Log_2_FC value, COPD group versus Ctrl group. Larger node size indicates higher Log_2_FC value, color in red represents the upregulation in COPD, in blue represents the downregulation in COPD. **E)** Western blotting of cell lysis from 1-HAE, 16-HBE, BCiNS1.1 and A549 cells were probed with an anti-HIF-3α antibody.

To explore the cellular localization of HIF-3α, we next using single cell RNA sequencing (scRNA-seq) data (GSE171541) from GEO. After dimensional reduction and annotation by Seurat and Celldex packages, we identified 7 cell types in the dataset (Figure 2A). To further evaluate the expression differences of HIF-3α among these 7 cell types, we compared the HIF-3α average expression value in both COPD and control groups (Figure 2C). Result shows, while HIF-3α is highly expressed in endothelial cells, it has nearly no changes between COPD and Control groups (Figure 2C). The absolute downregulation difference is greatest in epithelial cells (Figure 2D). To continually explore which subtype of lung epithelial cell express HIF-3α, we performed a multi-cell line verification. Although human lung is most often described as a dichotomous branching system of approximately 22 generations, the lung epithelial cells can be divided by 1) upper airway epithelial cells for gas conducting, 2) alveolar epithelial cells for respiration and 3) the middle transitional bronchioles 16. We further detected the HIF-3α expression in upper airways by including human airway epithelial (1-HAE) cell line and human bronchial epithelial (16-HBE) cell line; in alveolar epithelial cells, we included A549 cell line; in transitional bronchioles, we included human bronchial epithelial cells (BCiNS1.1) and cultured in air-liquid interface (ALI) condition. Result shows, HIF-3α has no expression in upper airway epithelial cells but is strongly expressed in the lower alveolar and ALI cell cultures (Figure 2E).

### HIF-3α expression and localization in COPD

**Figure 3:**
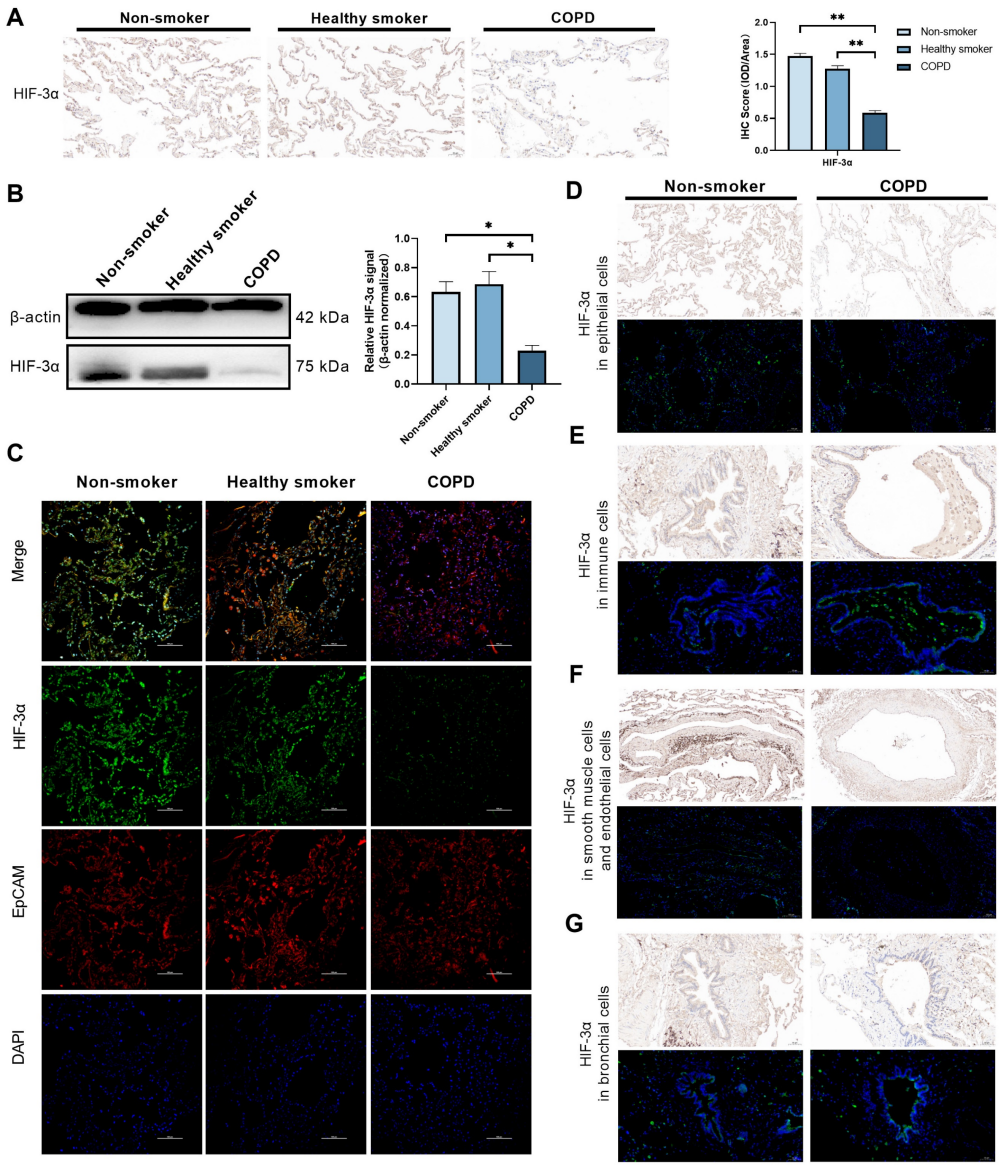
** HIF-3α expression and localization in protein level. A)** Immunohistochemistry showed that HIF-3α was markedly lower in COPD patients than smoker and non-smokers. Original magnification ×20. Scale bars: 50 μm. **B)** Western blotting of human lung homogenates from COPD, smokers and non-smokers were probed with an anti-HIF-3α antibody (normalized to β-actin). **C)** Co-localization of HIF-3α and EpCAM in human lung samples; Original magnification ×10. Scale bars: 100 μm. **D)** Immunohistochemistry and immunofluorescence showed the HIF-3α staining in epithelial cells. Original magnification ×10. Scale bars: 100 μm. **E)** Immunohistochemistry and immunofluorescence showed the HIF-3α staining in immune cells. Original magnification ×20. Scale bars: 50 μm. **F)** Immunohistochemistry and immunofluorescence showed the HIF-3α staining in smooth muscle and endothelial cells. Original magnification ×10. Scale bars: 100 μm. **G)** Immunohistochemistry and immunofluorescence show the HIF-3α staining in bronchial structures. Original magnification ×20. Scale bars: 50 μm. IOD: integrated optical density. *: p < 0.05; **: p < 0.01; ***: p < 0.005; ****: p < 0.001.

By far, we have shown HIF-3α mRNA level is downregulated in epithelial cells among COPD lungs and especially in alveolar epithelial cells. To test this phenomenon in protein level, we performed Immunohistochemistry and Western blots on an independent human lung sample, which consisting lungs from non-smokers (n = 5), smokers (n = 5) and COPD patients (n = 6) (Table 2). Result shows, HIF-3α protein level is significantly reduced in COPD lungs compared to those of non-smokers or smokers (Figure 3A-B). We further tested the localization of HIF-3α in epithelial cells by co-staining with EpCAM, which is widely used as the marker of epithelial cells 17. Result shows HIF-3α and EpCAM is co-localized in the COPD lungs (Figure 3C) and in the “COPD” mice (Figure S4) (i.e. mice exposed to CS for six months 18-20, the H&E staining and spirometry test of “COPD” mice were shown in (Figure S5). Besides, we also performed HIF-3α immunochemistry and immunofluorescence staining on series sections in nonsmoker and COPD patients respectively. Align with scRNA-seq data, these results suggested HIF-3α is downregulated in lung epithelial cells and basically in alveolar epithelial cells (Figure 2E, 3D) in protein level. Moreover, HIF-3α is upregulated in immune cells (Figure 3E), decreased in endothelial cells (Figure 3F), scarcely expressed in basal cells among bronchial structures (Figure 3G) and is no staining in smooth muscle cells (Figure 3F). Based on emphysema is characterized by the death of alveolar epithelial cells and is the hall marker of COPD, we next focused on alveolar epithelial cells.

### Bulk-RNA seq of HIF-3α overexpression cells revealed ferroptosis

**Figure 4:**
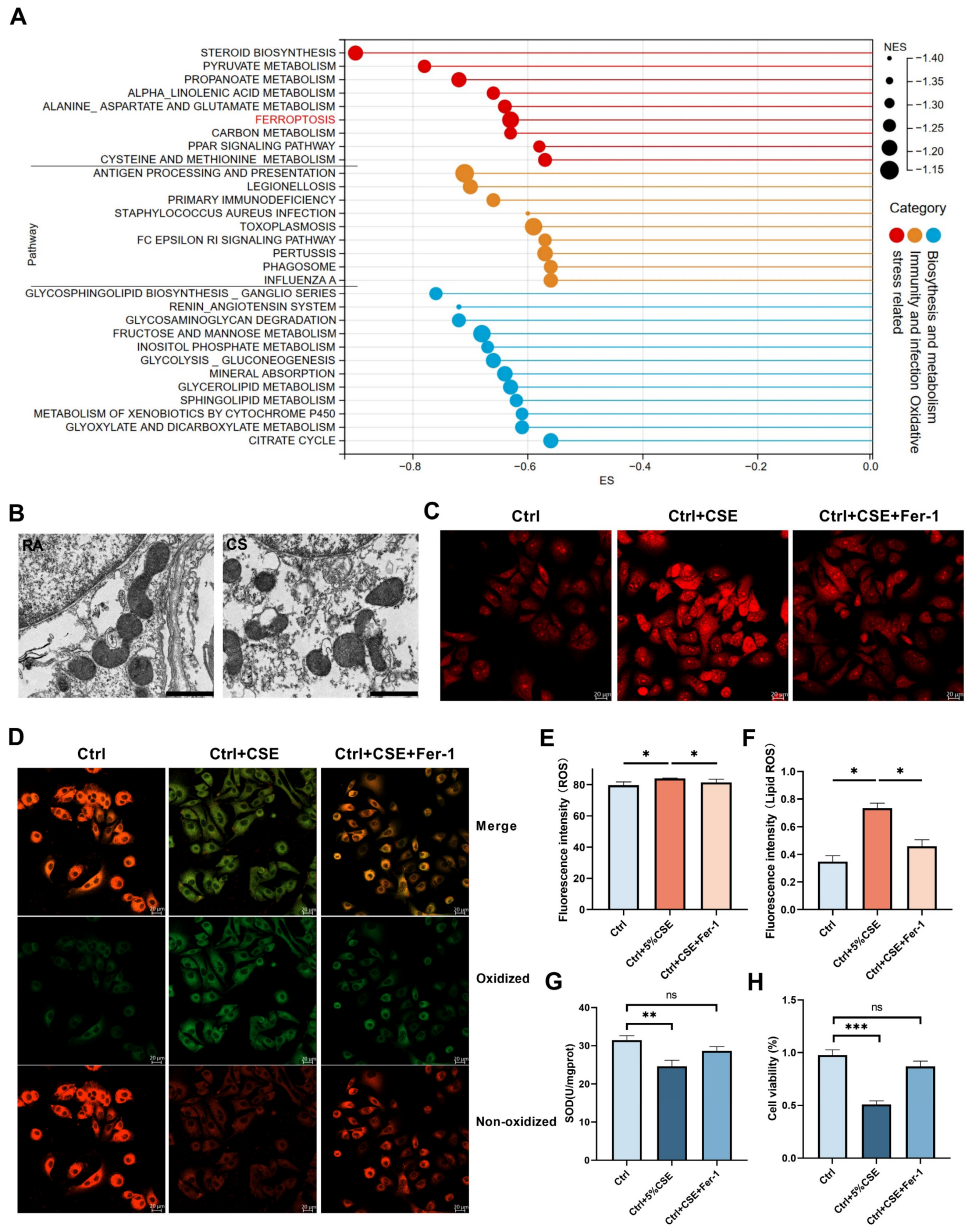
** Involvement of ferroptosis in COPD. A)** Pathways generated in GSEA analysis. Larger size indicates higher normalized enrichment scores (NES). **B)** Representative transmission electron microscopy (TEM) images of six months mice model lung. Room air (RA), cigarette smoke (CS); scale bar 1μm. **C, E)** Images of intracellular ROS stained by DCFH-DA were captured using confocal fluorescence microscope in MLE-12 cells. Scale bars: 20 μm. **D, F)** Images of Lipid ROS stained by C11-BODIPY 581/591 were captured using confocal fluorescence microscope in MLE-12 cells. Scale bars, 20 μm. **G)** Superoxide dismutase (SOD) levels in MLE-12 cell models. **H)** Cell viability of each group was analyzed by the CCK-8 assay and all values were normalized to those of the control group. IOD: integrated optical density. *: p < 0.05; **: p < 0.01; ***: p < 0.005; ****: p < 0.001. ns: p > 0.05 (nonsignificant).

To understand the potential mechanisms by which HIF-3α contributes to oxidative stress, and for the consistency of following* in vivo* animal experiments, we choose murine lung alveolar epithelial (MLE-12) cells for following lentivirus transfection and bulk-RNA seq (Figure S6A-B). Western blot and volcano plot showed that this approach led to HIF-3α overexpression (Figure S6A, C). We performed gene set enrichment annotation (GSEA) analysis on this RNA-seq data and filtered the pathways based on |NES|>1, p<0.05, FDR q-value<0.25. According to the criteria above, we obtained and divided the pathways into three clusters (Figure 4A): “oxidative stress related”, “immunity and infection” and “biosynthesis and metabolism” 21-24. Among these pathways, ferroptosis attracted our attention as it is characterized by lipid peroxidation and is a subtype of oxidative stress in COPD (Figure S6D) 25,26.

### Involvement of ferroptosis in COPD

Ferroptosis is a regulated cell death, which is induced by lipid peroxidation and ROS 25,26. On transmission electron microscopy, ferroptosis character is shown as dense and smaller mitochondria with increased membrane density and vestigial cristae 27. We evaluated the lung tissue from the COPD mice using transmission electron microscopy to detect these morphological changes. The mitochondria in the lungs of the COPD mice were much denser and shrunken compared to those in the control group (Figure 4B). Biochemically, ferroptosis is characterized by higher level of lipid peroxidation and ROS. By using C11-BODIPY 581/591 and ROS probe, we found the lipid peroxidation (Figure 4D, F) and ROS levels (Figure 4C, E) were significantly augmented in the CSE exposed cells and could be inhibited by Fer-1, which is widely used as the inhibitor of ferroptosis 26. SOD (superoxide dismutase) is an important antioxidant and prevents ROS-initiated reactions 28. Thus, we measured SOD levels in mice lungs (Figure S7A) and COPD lungs (Figure S7B). We also tested SOD levels (Figure 4G) and cell viability (Figure 4H) in CSE treated cells. Glutathione peroxidase 4 (GPx4) has been reported as a significant anti-oxidation modulator in ferroptosis 27. In this current study, we found GPx4 is significantly lower in COPD patients than in non-smokers and smokers by immunohistochemistry (Figure S7D) and by western blotting (Figure S7E) (Table 2). This phenomenon was further shown in COPD mice samples (Figure S7C). Consistent with previous studies 29, ferroptosis is significantly increased in COPD and is reproducible in our experimental conditions.

### HIF-3α promote GPx4 expression and alleviate the ferroptosis in COPD

**Figure 5:**
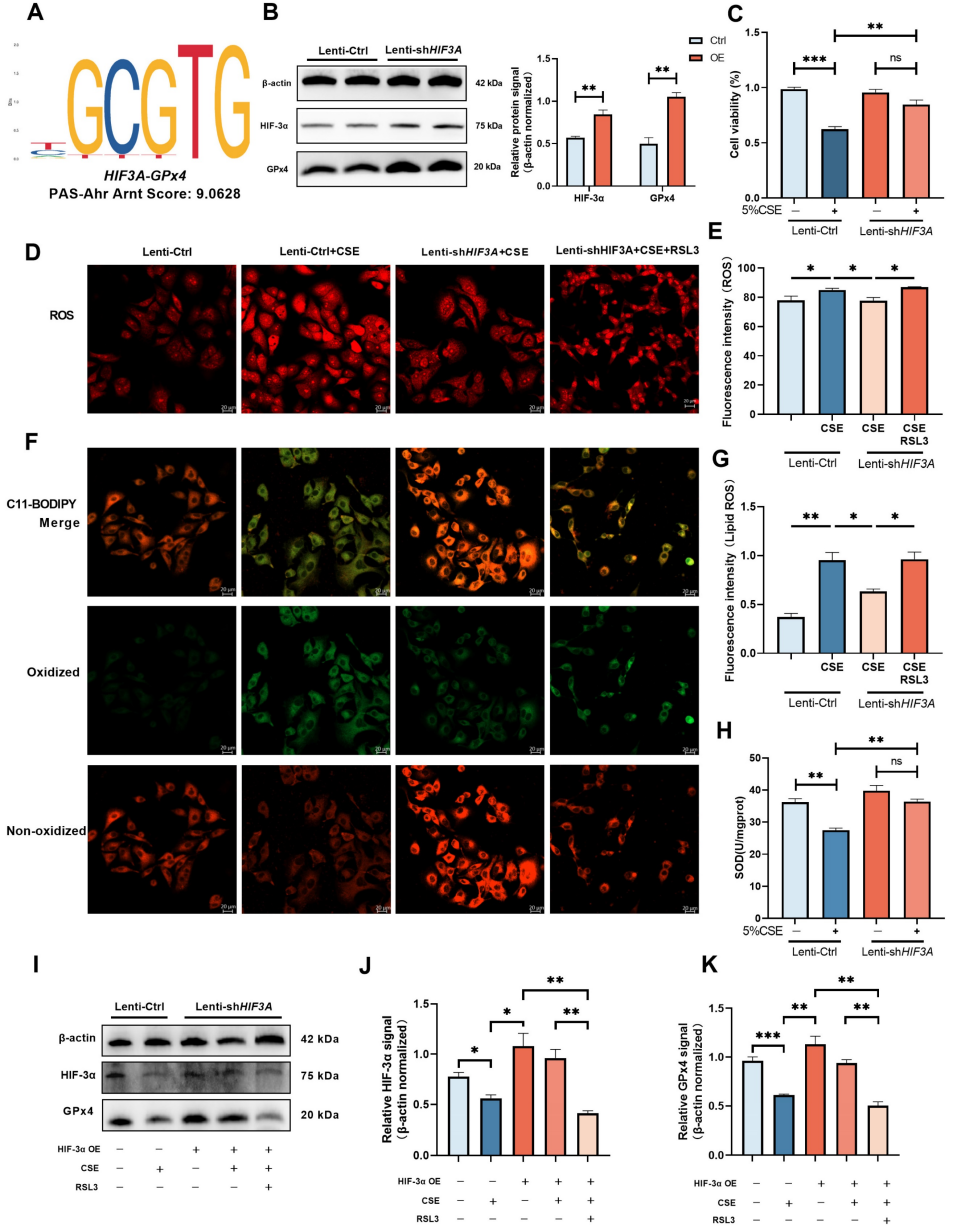
** Overexpression of HIF-3α activate GPx4 and alleviate the ferroptosis in COPD. A)** JASPAR prediction result shows HIF-3α shares a specific motif with GPx4. **B)** HIF-3α protein was detected by Western blot and normalized to β-actin in MLE-12 alveolar epithelial cells. **C)** Cell viability of each group was analyzed by the CCK-8 assay and all values were normalized to those of the control group. **D-E)** Images of intracellular ROS stained by DCFH-DA were captured using confocal fluorescence microscope in MLE-12 epithelial cells. Scale bars: 20 μm. **F-G)** Images of Lipid ROS stained by C11-BODIPY 581/591 were captured using confocal fluorescence microscope in MLE-12 epithelial cells. Scale bars, 20 μm. **H)** SOD levels detected in each group. **I-K)** Lenti-Ctrl and Lenti-shHIF3A cells were treated with RSL3 and CSE separately. HIF-3α and GPx4 were detected by western blotting and normalized to β-actin. *: p<0.05; **: p<0.01; ***: p<0.005; ****: p<0.001. ns: p>0.05 (nonsignificant).

Previous studies have demonstrated HIF-3α is a transcript activator that can regulate a distinct transcriptional response under hypoxia via its basic-helix-loop-helix and Per/Arnt/Sim regions (N-terminus) and transactivation domain (N-terminus) 14,15,30. To evaluate the possible association between HIF-3α and GPx4, we explored transcription factor binding sites by JASPAR database 31. HIF-3α was found to share a specific transcription motif with GPx4 (Figure 5A); HIF-3α overexpression significantly promoted the GPx4 expression in both CSE untreated (Figure 5B) and CSE treated (Figure 5I) cells. Overexpression of HIF-3α opposed the reduction of cell viability (Figure 5C) and SOD levels (Figure 5H), prevented the increasement of ROS (Figure 5D-E) and lipid peroxidation (Figure 5F-G). These findings indicated that overexpression of HIF-3α prevents the depletion of key anti-oxidation factors such as GPx4 and SOD, and further inhibits the alveolar epithelial cells ferroptosis.

### GPx4 is the major functional factor of HIF-3α

To explore the role of GPx4 underlying the overexpression of HIF-3α, we blocked GPx4 with RSL3 (Figure 5I) 27,32. In the presence of GPx4 inhibition, overexpression of HIF-3α is failed to reverse the high ROS (Figure 5D-E) and lipid peroxidation caused by CSE stimulation (Figure 5F-G). Which suggest GPx4 is the major anti-oxidative factor under the overexpression of HIF-3α and the presence of the HIF-3α-GPx4 axis.

### *Sftpc^Creert2/+^R26^LSL-Hif3a^* knock-in prevents lung function decline in 6-months smoking

**Figure 6:**
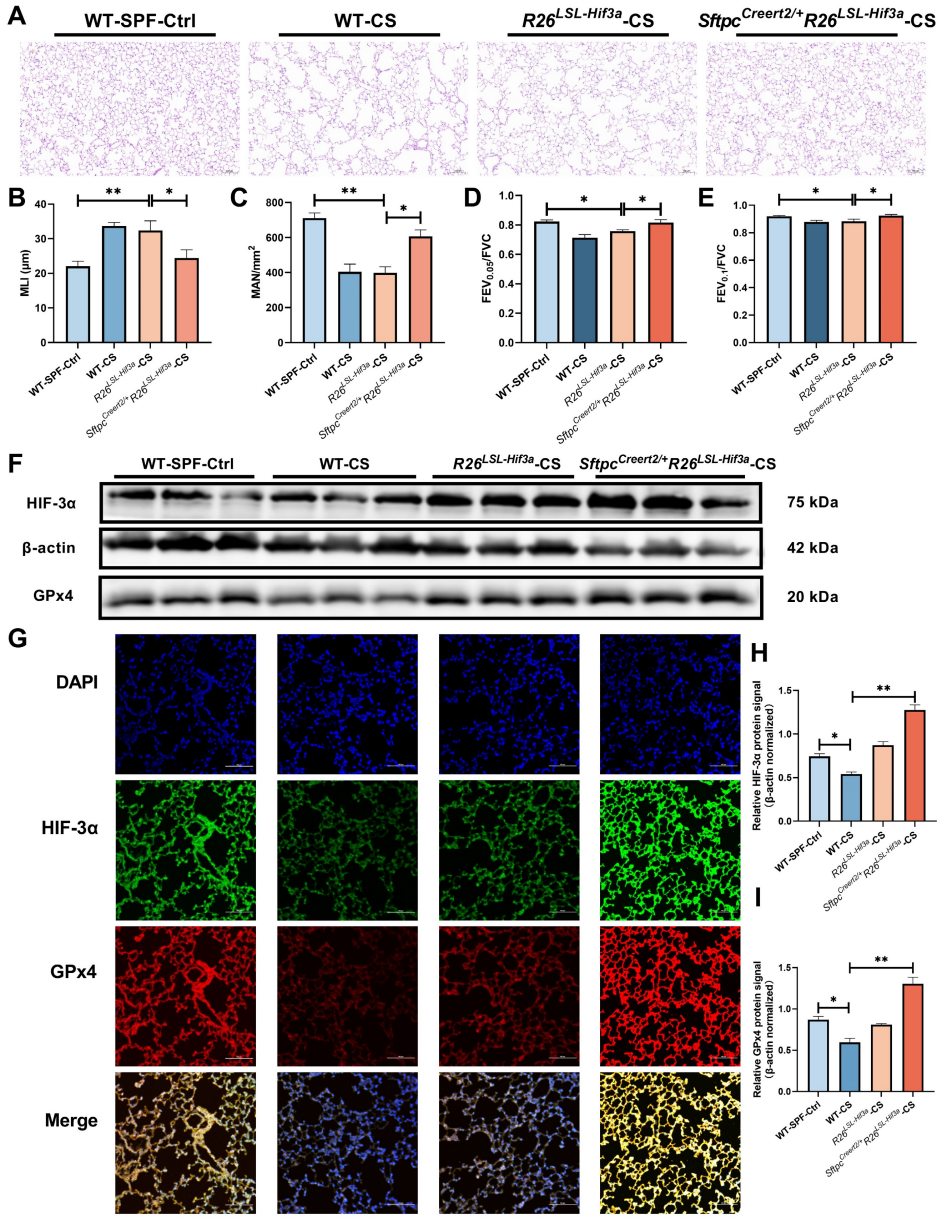
** Sftpc^Creert2/+^R26^LSL-Hif3a^ knock-in prevent lung function decline in 6-months smoking. A)** Result of H&E staining in mice model lung specimens were shown. Original magnification ×20. Scale bars: 50 μm. Wild type (WT); cigarette smoking (CS). We further evaluated the **B**) mean linear intercept (MLI) and **C**) Mean Alveolar Number (MAN) in each group. Using a FlexiVent system, we tested the **D**) FEV_0.05_/FVC, **E**) FEV_0.1_/FVC for each mouse at least three times. **F, H-I)** Western blotting of mice lung homogenates from each group were probed with an anti-HIF-3α antibody or with an anti-GPx4 antibody (normalized to β-actin). **G**) immunofluorescence staining for HIF-3α and GPx4 in each group were shown. Original magnification ×10. Scale bars: 100 μm. *: p < 0.05; **: p < 0.01.

By far, we have elucidated overexpression of HIF-3α can alleviate alveolar epithelial cells ferroptosis in COPD, and the mechanism is mainly depended on the activated HIF-3α-GPx4 axis *in vitro*. To test this mechanism *in vivo*, we further constructed alveolar epithelial cells specified *Sftpc^Creert2/+^R26^LSL-Hif3a^* knock in mice, and exposed them for 6-months cigarette smoking. H&E staining shows, *Sftpc^Creert2/+^R26^LSL-Hif3a^* group has much better mean liner intercept (MLI) and mean alveolar number (MAN) compared with *R26^LSL-Hif3a^* group, suggest the higher level of lung integrity, alveolar numbers and less presence of emphysema (Figure 6A-C). Besides, we also measured the lung function for each group, notably, *6-months smoking Sftpc^Creert2/+^R26^LSL-Hif3a^* group has a much higher lung function results, as shown by FEV_0.05_/FVC and FEV_0.1_/FVC (Figure 6D-E). We further tested the HIF-3α and GPx4 expression by western blot and immunofluorescence staining, result shows HIF-3α and GPx4 is significantly upregulated in *Sftpc^Creert2/+^R26^LSL-Hif3a^* group (Figure 6F-G). Results demonstrated the activation of the HIF-3α-GPx4 axis was able to prevent the decline of lung function in vivo.

### *Sftpc^Creert2/+^R26^LSL-Hif3a^* knock-in alleviate alveolar epithelial cells ferroptosis

**Figure 7:**
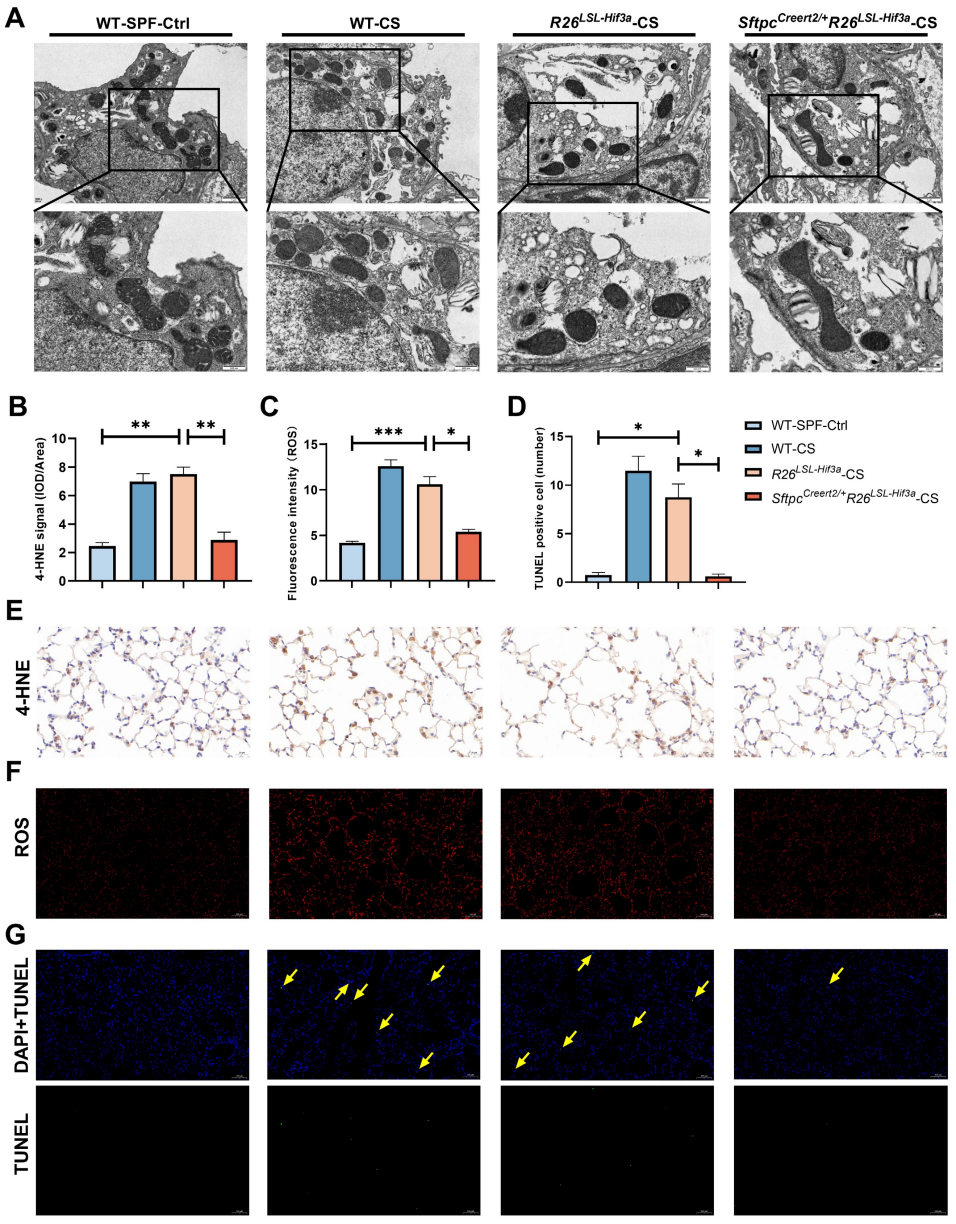
** Sftpc^Creert2/+^R26^LSL-Hif3a^ knock-in alleviate ferroptosis in 6-months smoking. A)** Representative transmission electron microscopy (TEM) images of six months mice model lung; scale bar 1μm, 500nm. **B**) Statistical plot of 4-HNE, **C)** ROS and **D)** TUNEL results. **E)** Immunohistochemical staining of 4-HNE in mice lung. Scale bars: 20μm. **F**) immunofluorescence staining of intracellular ROS in mice lung. Scale bars: 100μm. **G)** TUNEL assay staining (green) in mice lung. Nuclei were counterstained with DAPI (blue). Positive cells were marked with yellow arrows. Scale bars: 100μm. *: p < 0.05; **: p < 0.01; ***: p < 0.001.

We next examined the morphological characteristics of mitochondria in alveolar epithelial cells under TEM. Compared with WT-SPF group, we observed much smaller and shrunken mitochondria in cigarette treated wild type group (WT-CS) and negative control (*R26^LSL-Hif3a^*) group. We also found the disrupted mitochondria cristae and increased membrane density in WT-CS group. These phenomena were shown to be alleviated in *Sftpc^Creert2/+^R26^LSL-Hif3a^*-CS group with larger mitochondria and clear cristae (Figure 7A). After 6-month cigarette exposure, 4-HNE and ROS probe were used to evaluate the lipid peroxidation and oxidative level in each group, respectively. 4-HNE staining enhanced in WT-CS and *R26^LSL-Hif3a^* groups, especially in type Ⅱ pneumocytes and alveolar macrophages. Overexpression of HIF-3α in alveolar epithelial cells *in vivo* alleviate the peroxidation (Figure 7B, E) and ROS level (Figure 7C, F). We further measured the alveolar epithelial cells viability by using TUNEL staining. TUNEL positive cells were significantly increased in WT-CS and *R26^LSL-Hif3a^* groups, and this phenomenon was alleviated in *Sftpc^Creert2/+^R26^LSL-Hif3a^*-CS group (Figure 7D, G). Collectively, the activation of the HIF-3α-GPx4 axis prevents COPD by inhibiting alveolar epithelial cell ferroptosis.

## Discussion

COPD patients are largely asymptomatic or minimally symptomatic until the disease progresses to advanced stages when the prognosis is generally poor. Thus, it is not appropriate to confine the research to clinical symptoms, pathological lesions like emphysema, small airway disease and the risk factor of smoking should be included. In this study, we performed bulk-RNA sequencing and a series of experiments on human lung samples from nonsmokers, smokers, pre-COPD, and COPD patients. In contrast to the traditional concept of high-level oxidative stress in COPD patients, we identified the peak of ROS levels occurring in pre-COPD samples, suggesting oxidation-related damage begins years before the onset of COPD. Besides, by leveraging the pseudo-time algorithm to analyze the bulk-RNA sequencing data, we identified the core gene as HIF-3α.

Using single-cell RNA sequencing data complemented by *in vivo* and *in vitro* experiments, we found that HIF-3α was largely downregulated in lung epithelial cells, especially in lower alveolar epithelial cells and transitional bronchioles. Notably, HIF-3α is not expressed in upper airway epithelial cells which functioned as gas conducting, suggesting the upper epithelial cells and alveolar epithelial cells have two distinct patterns in response to oxygen changes and have different sources of oxygen supply.

Under normoxia, HIF-α subunits are continuously hydroxylated by the von Hippel-Lindau (VHL) protein due to the shared common oxygen-dependent degradation (ODD) domain 33,34. Thus, in steady-state HIF-α cannot accumulate, a process catalyzed by prolyl hydroxylase (PHD), a cellular sensor for low oxygen 35. In hypoxic conditions, when PHD is inhibited, the HIF-α level increases rapidly. In the HIF family, three factors, HIF-1α, HIF-2α and HIF-3α have been identified. Among them, HIF-3α is the most recently identified and the least investigated factor 14. HIF-1α and HIF-2α as the master in response to hypoxia, and HIF-3α with the limited function mainly featured as the negative regulator of HIF-1α and HIF-2α 36,37. However, recent studies demonstrated a distinct sequence and transcript function for HIF-3α compared to HIF-1α/2α.

Gu et al. showed that HIF-3α had a relatively low sequence identity with HIF-1α/2α 38. HIF-1α and HIF-2α have two transactivation domains (TAD) at N and C-termini, while only one TAD at the N-terminus for HIF-3α that shares 58% identity with the N-TAD in HIF-1α, 52% identity with the N-TAD in HIF-2α 38,39. Besides, HIF-3α has an evolutionarily conserved unique leucine zipper domain (LZIP) and an LXXLL 40. Importantly, Zhang et al. revealed that HIF-3α exhibits significant transactivation activity in zebrafish and identified a group of gene that are upregulated by HIF-3α only 15. These observations suggested that HIF-3α functions as a complementary regulator with HIF-1α/2α and is involved in the response to hypoxia. Currently, it is widely accepted that HIF-3α has dual functions: Inhibition of HIF-1α and HIF-2α activities, and transcriptional regulation of its target genes 14,15 to further influence oxygen tension changes.

We noticed that HIF-3α mRNA levels were upregulated up to the pre-COPD stage (patients with emphysema but no airflow limitation). In the COPD stage, the HIF-3α mRNA level was dramatically downregulated accompanied by the upregulation of HIF-1α and HIF-2α (Figure S8). However, the reason for the HIF-3α downregulation in COPD patients is still unclear. Chen et al conducted a systemic hypoxia exposure experiment on rats at 10.0% ± 0.5% O_2_ for 3, 7, 14, and 21 days. The results showed an elevation of HIF-3α expression during systemic hypoxia, decreasing slowly after 14 days, accompanied by increased HIF-1α and HIF-2α expression in lung tissues 41, an observation that was consistent with our study. The transient elevation of HIF-3α could be explained by the inhibition of PHD and VHL under hypoxia. However, in prolonged chronic hypoxia, which often occurs in COPD, one possible reason for downregulated HIF-3α is its competition with HIF-1α and HIF-2α in binding to the HIF-1β subunits, depleting HIF-3α in COPD 14.

ROS is also an important signaling molecule 42,43, especially mitochondria-derived ROS (mtROS) mediates diverse signaling pathways to provoke immune responses, elicit antioxidant pathways, and regulate ferroptosis 44. mtROS can inhibit the activity of PHD and thus enhance HIF-α expression 45. Mansfield et al. showed Cytochrome c null cells exhibited attenuated ROS production under hypoxia compared to WT cells, and the Cytochrome c null cells with Complex III inhibitor myxothiazol elicited no change in ROS production, while the inhibitor-treated WT cells showed decreased ROS production under hypoxia conditions. The results suggested the mitochondrial origin of ROS under hypoxia, and mtROS generated by Complex Ⅲ is essential for hypoxic HIF-α stabilization 46.

In this study, we found that HIF-3α expression was also downregulated in the presence of GPx4 inhibition. GPx4, members of the glutathione peroxidase family, could detoxify the lipid hydroperoxides to lipid alcohol by catalyzing glutathione reduction, and thus, restoring mtROS homeostasis 46. These results suggested mutual regulation between mtROS and HIF-3α. Collectively, the transition expression pattern of HIFs ranging between highly expressed HIF-3α and low HIF-3α expression accompanied by HIF-1α/2α upregulation suggests the cells are subjected to severe hypoxia pressure. The significant imbalance of mtROS, which leads to COPD in reversible airflow limitation, severe hypoxia and hypoxemia. Thus, the focus should be on identifying and implementing interventions in the pre-COPD stage in the clinic, as this may be the last available stage to improve the prognosis effectively.

Our data are consistent with previous studies, demonstrating the involvement of ferroptosis and its regulator, GPx4 in the pathogenesis of COPD 25,28. We also showed that HIF-3α was downregulated in COPD lungs which was in line with two previous studies 48,49. It is of note that in one study, which used a CS-induced mouse model, HIF-3α was upregulated in the lungs at 15 weeks of CS exposure 50. However, the standard exposure of CS to induce COPD-like changes in the lungs of mice is 6 months 19,20. In our 6-month CS mouse model, we observed a significant downregulation of HIF-3α expression (Figure S9).

As is the case with multi-stage pathology-related research investigations, our study has certain limitations. Ideally, tissue sampling should take place over time to fully observe the structural and molecular changes especially in pre-COPD that occur with CS. Second, the transcriptomics toward the nonsmokers, smokers, pre-COPD, and COPD patients may not entirely represent the pathological process. In future studies, genomics, epigenomics, and proteomics in small airway disease and GOLD classification should be included. Finally, the transcript activation role of HIF-3α in targeting GPx4 needs to be validated using chromatin immunoprecipitation (ChIP) in future.

Chronic diseases like COPD are largely asymptomatic in the early stages. However, the intracellular homeostatic system is imbalanced and compromised at the molecular level. By dividing the decades-long pathological process into etiology, pathology and clinical manifestations, we found that ROS was enriched in the pre-COPD stage, and the transition expression pattern of HIFs from pre-COPD to COPD potentially aggravated COPD. Therefore, special attention should be directed to the pre-COPD stage in future clinical and experimental studies.

By leveraging this comprehensive analysis, more promising treatments, targets will be discovered and enhance our understanding of the molecular pathogenesis of the chronic diseases like COPD.

## Materials and Methods

### Participants and ethics approval / Bulk-RNA sequencing

52 Lung specimens were obtained from patients who underwent pulmonary resection surgery at the China-Japan Friendship Hospital. Among them, 36 lung samples were distributed equally across the 4 groups: 1) non-smokers, 2) smokers, 3) pre-COPD patients and 4) COPD patients based on the GOLD criteria 1. Briefly, pre-COPD was defined as current or former smokers who had a post-bronchodilator FEV_1_/FVC of ≥ 0.7, combined with a diffusing capacity of the lung for carbon monoxide (DLco) of <80% predicted and/or the presence of emphysema on computed tomography (CT) 1,6. COPD was defined as current or former smokers with a post-bronchodilator FEV_1_/FVC of < 0.7. Patients with a history of asthma were excluded. The remaining 16 lung samples were used as independent group for verification and mechanism exploring. Tissue characteristics were determined by a histopathological evaluation of paraffin-embedded sections. Approval for this study was granted by the Medical Science Research Ethics Committee of China-Japan Friendship Hospital (2019-106-K74), and all participants gave informed consent.

After RNA extraction from tissue samples, its integrity was assessed using a Bioanalyzer 2100 system RNA Nano 6000 Assay Kit (Agilent Technologies, CA, USA). Poly-T oligo attached magnetic beads were then used to purify mRNA from total RNA. Fragmentation was carried out using divalent cations under elevated temperature in a First Strand Synthesis Reaction Buffer (5X). Followed by first and second strand cDNA synthesis. AMPure XP system (Beckman Coulter, Beverly, USA) was used to select the length of cDNA in 370~420 bp. PCR amplification with purified by AMPure XP beads was then used to generate the library.

### Mice

The *Sftpc^Creert2/+^R26^LSL-Hif3a^* knock-in mice were generated at the Shanghai Model Organisms Center, Inc. Briefly, using CRISPR/Cas9 method to insert the CAG-LSL-Hif3a-WPRE-pA expression cassette into the Rosa26 gene site through homologous recombination. Obtain Cas9 mRNA and gRNA through in vitro transcription; construct the donor vector through In-Fusion cloning. The donor vector contains a 3.3 kb 5' homology arm, CAG-LSL-Hif3a -WPRE-pA and 3.3 kb 3' homology arm. Cas9 mRNA, gRNA and donor vector were microinjected into the oocyte of the C57BL/6J mice to obtain the F0 generation mice. The F0 generation mice identified as positive by PCR amplification and sequencing were mated with C57BL/6J mice to obtain 3 positive F1 generation mice. All mouse strains were genotyped by PCR using the following primers: wild-type allele (941 bp), P1: 5'-TTTGCTACTAACTTCCCATGGCT-3', P2: 5'-TCCAACTCTGCCTATTCACCG-3', knock-in allele (396 bp), P3: 5'-TCTGCAAGGTCGACAACTCC-3', P4: 5'-CGGGCCACAACTCCTCATAA-3'.

### Pseudo-time analysis and core gene identification

For the 36 human lung samples Bulk-RNA sequencing data, a fuzzy c-means algorithm of the Mfuzz package (2.58) in R was applied to cluster the genes according to their dynamic expression patterns 51. The average reads per kilobase million (RPKM) values for each gene across the 4 groups were used as the input data and assigned each gene to a specific cluster based on its expression value in all 4 groups after standardisation.

### Immunohistochemistry (IHC) for lung sections

Samples of lung tissue were fixed in a 4% paraformaldehyde solution (Beyotime, Nantong, China) for 24 h and then dehydrated, embedded in paraffin and sectioned using routine methods 25. The sections were then incubated overnight at 4°C with anti-HIF-3α (1:100 dilution; Proteintech, Wuhan, China), anti-GPx4 (1:1000 dilution; Abcam, UK) or 4-HNE (Bioss, Beijing, China) antibodies. They were subsequently incubated with anti-horseradish peroxidase (HRP) antibody (1:200 dilution, SeraCare, Beijing, China) at room temperature for 30 min. At least five optic planes were taken from each slice for evaluation. Using Image-Pro Plus to measure the integrated optical density (IOD).

### Cigarette smoke exposure

Wild type C57BL/6 mice (8-10 weeks, female, weighting 22 ± 2 g) were purchased from Beijing Sipeifu Biotechnology Co., Ltd., China. Cigarette smoking (CS) group mice were placed in a whole-body exposure chamber and exposed to tobacco cigarette smoke (Marlboro™, four times/day and ten cigarettes/time for five days/week) 18,52. The mice in the control group were exposed to room air (RA). The total particulate matter (TPM) concentrations were maintained at 160-180mg/m^3^, as measured using an Aerosol Monitor (pDR-1500, Thermo Scientific™, USA).

### Mice lung function measurements

Mice lung function was measured by FlexiVent system (Scireq, Montréal, Canada). The mice were anesthetized with 2% pentobarbital sodium (0.1ml/10g), tracheostomized, and then connected to the FlexiVent system. The tidal volume and breathing frequency were set at 10 ml/kg and 150 breaths/min respectively 18.

### Western blotting

Procedure of protein lysis and Western blotting were based on a previously method 25. Briefly, after 1 h membrane blocking, they were incubated overnight at 4°C with anti-HIF-3α (ABclonal, Wuhan, China), anti-GPx4 (1:1000 dilution; Abcam, UK), and anti-β-actin (ABclonal) antibodies. Using automatic exposure machine (ProteinSimple, Minneapolis, MN, USA) to develop the bands.

### scRNA-seq analysis and HIF-3Α localization exploration

The scRNA-seq dataset (GSE171541) for COPD can be accessed in the Gene Expression Omnibus (GEO) database; it contains sequencing data of 110,931 cells, which were extracted from peripheral lung tissues of 9 patients; the original patient demographic information has been previously reported 53. The samples were integrated with anchors using the Seurat package of R 54. The t-SEN and UMAP 55 packages were used to reduce dimensionality to 2-or 3-dimensions. The singleR package of R 56 and Human Primary Cell Atlas Data were used to annotate different cell types; data pertaining to HIF-3Α expression levels in the different cell types of the control and COPD groups were then extracted and analysed.

### Cell culture experiments

Human airway epithelial (1-HAE) cells, human bronchial epithelial (16-HBE) cells, and murine lung epithelial (MLE-12) cells were cultured using DMEM medium (Gibco, Grand Island, NY, USA) with 10% FBS, 100 IU streptomycin and 100 IU penicillin in an incubator in 37 °C with 5% CO2. For human bronchial epithelial cells (BCiNS1.1) were cultured in air liquid interface (ALI) using PneumaCult™-Ex and PneumaCult™-ALI (STEMCELL Technologies, Vancouver, CANADA). Cigarette smoke extract (CSE) was prepared based on a previously published method 57. Briefly, one 3R4F reference cigarette containing 9.4 mg tar and 0.73 mg nicotine (University of Kentucky, USA) was bubbled into 10 mL of a high-glucose DMEM (Gibco); this was subsequently diluted to a concentration of 5% for use. According to experiments, cell lines were pretreated for 30 min or treated for 24 h with 0.1 μM RSL3 (HY-100218A, MCE, USA), 1 μM Fer-1 (HY-100579, MCE, USA) or 5% CSE.

### Transmission electron microscopy

Fresh mice lung specimens were thinly sliced and rapidly fixed and dehydrated using a gradient series of acetone. The sections were then stained and examined using a transmission electron microscope (HT7800; Hitachi, Japan).

### Lipid peroxidation and TUNEL analysis

Lipid peroxidation and Intracellular reactive oxygen species (ROS) levels were measured using 2 ′,7 ′-dichlorofluorescein diacetate (DCFH-DA, Beyotime, China) and the BODIPY 581/591 C11 probe (C10445, Invitrogen, MA, USA), respectively, according to the manufacturer's instructions. MLE-12 cells were cultured into six-well plates and cultured for 24 h to ensure adherence. Following appropriate treatment, the cells were stained with prewarmed 2′,7′-dichlorofluorescein diacetate (5 μM) or C11-BODIPY (10 μM) probe; they were then incubated at 37 °C for 30 min in dark with fetal bovine serum-free medium. Using in situ cell death detection kit (11684817910, Roche, Basel, Switzerland) to perform the TUNEL assay according to manufacturer's instruction. The cells were rinsed thrice and the images were acquired using a LSM900 Zeiss confocal laser scanning microscope (Zeiss, Germany).

### Statistical analysis

Data were expressed as the means ± SEM taken from at least three independent experiments unless otherwise stated. Statistical analyses were performed using GraphPad Prism 8 software (San Diego, CA, USA) and SPSS 21.0 software (IBM, NY, USA), with *p*<0.05 considered as significant. Statistical differences were determined by an unpaired Student's t test or by one-way analysis of variance with Bonferroni correction. Pearson's correlations were calculated between two parameters.

## Supplementary Material

Supplementary figures.

## Figures and Tables

**Table 1:** Clinical Characteristics of Enrolled Subjects for RNA-bulk Sequencing

	Non-smokers (n = 9)	Smokers (n = 9)	Pre-COPD smokers (n = 9)	COPD smokers (n = 9)
Age (years)	56.3 ± 4.8	55.8 ± 6.6	60.4 ± 3.0	61.2 ± 4.2
Sex (male)	4/9	5/9	5/9	6/9
FEV1 % pred	94.4 ± 9.7	100.7 ± 9.1	97.2 ± 7.8	69.6 ± 18.4*^##+^
FEV_1_/FVC	81.0 ± 4.9	76.8 ± 4.2	75.4 ± 1.6	58.0 ± 5.9**^#+^
RV/TLC	31.67 ± 4.0	38.07 ± 1.9	41.70 ± 2.7**^#^	43.11 ± 7.3**
Pack/year	0	30.5 ± 24.6	20.0 ± 15.5	38.9 ± 43.3
Current/Former Smokers	0/0	5/4	4/5	2/7

**COPD:** chronic obstructive pulmonary disease; **FEV_1_:** forced expiratory volume in one second; **FVC:** forced vital capacity, **FEV1 % pred** and **FEV_1_/FVC** were measured after post bronchodilator.** RV:** residual volume; **TLC:** total lung capacity. Data are presented as means ± S.D. Descriptive data for the intervention and comparison groups were compared using the chi-square test for categorical variables and ANOVA for quantitative data. *p < 0.05 versus non-smokers, **p < 0.01 versus non-smokers. ^#^p < 0.05 versus smokers, ^##^p < 0.01 versus smokers. ^+^p < 0.05 versus pre-COPD.

**Table 2:** Clinical Characteristics of Enrolled Subjects for IHC, IF and WB experiments

	Non-smokers (n = 5)	Smokers (n = 5)	COPD smokers (n = 6)
Age (years)	50.6 ± 10.3	43.8 ± 7.9	58.8 ± 7.4
Sex (male)	2/5	4/5	4/6
FEV1 % pred	104.1 ± 16.3	97.0 ± 3.3	70.9 ± 18.9*^#^
FEV_1_/FVC	76.1 ± 4.8	74.6 ± 2.8	61.0 ± 7.7*^#^
Pack/year	0	26.8 ± 24.5	34.2 ± 17.2
Current / former smoker	0/0	1/4	3/3

**COPD**: chronic obstructive pulmonary disease; **FEV_1_:** forced expiratory volume in one second; **FVC:** forced vital capacity, **FEV1 % pred** and **FEV_1_/FVC** were measured after post bronchodilator. Data are presented as means ± S.D. Descriptive data for the intervention and comparison groups were compared using the chi-square test for categorical variables and ANOVA for quantitative data. *p < 0.05 versus non-smokers, ^#^p < 0.05 versus smokers.

## Abbreviations

COPD: Chronic Obstructive Pulmonary Disease
HIF-3α: Hypoxia Inducible Factor-3α
ROS: Reactive Oxygen Species
CSE: Cigarettes Smoking Extracts
GPx4: Glutathione Peroxidase 4
RPKM: Reads Per Kilobase Million
log_2_FC: Log_2_-Fold-Change
1-HAE: Human Airway Epithelial
16-HBE: Human Bronchial Epithelial
ALI: Air-Liquid Interface
EpCAM: Epithelial Cell Adhesion Molecule
H&E: Hematoxylin-Eosin
scRNA-seq: Single Cell RNA sequencing
MLE-12: Murine Lung Epithelial-12
GSEA: Gene Set Enrichment Annotation
NES: Normalized Enrichment Score
SOD: Superoxide Dismutase
MLI: Mean Liner Intercept
MAN: Mean Alveolar Number
WT: Wild Type
CS: Cigarette smoking
4-HNE: 4-Hydroxynonenal
TUNEL: TdT-mediated dUTP Nick-End Labeling
PHD: Prolyl Hydroxylase
ODD: Oxygen-Dependent Degradation
VHL: Von Hippel-Lindau
TAD: Transactivation Domains
LZIP: Leucine Zipper Domain
mtROS: Mitochondria-Derived ROS
ChIP: Chromatin Immunoprecipitation
IOD: Integrated Optical Density
TPM: Total Particulate Matter
RA: Room Air
GEO: Gene Expression Omnibus
