# Supplementary material for: Hypoxia inducible factor (HIF) 3α prevents COPD by inhibiting alveolar epithelial cell ferroptosis via the HIF-3α-GPx4 axis
Source: Theranostics. 2024 Aug 28;14(14):5512–27. doi: 10.7150/thno.99237 (PMC11413794; doi:10.7150/thno.99237)
Supplement: Supplementary file 1 — Supplementary figures. [file thnov14p5512s1.pdf]

Supplementary Materials for

- **Hypoxia inducible factor (HIF) 3α prevents COPD by inhibiting alveolar epithelial cell ferroptosis via the HIF-3α-GPx4 axis**

Junchao Jiang *et al.*

This PDF file includes:

Supplementary
Figs: S1 to S9

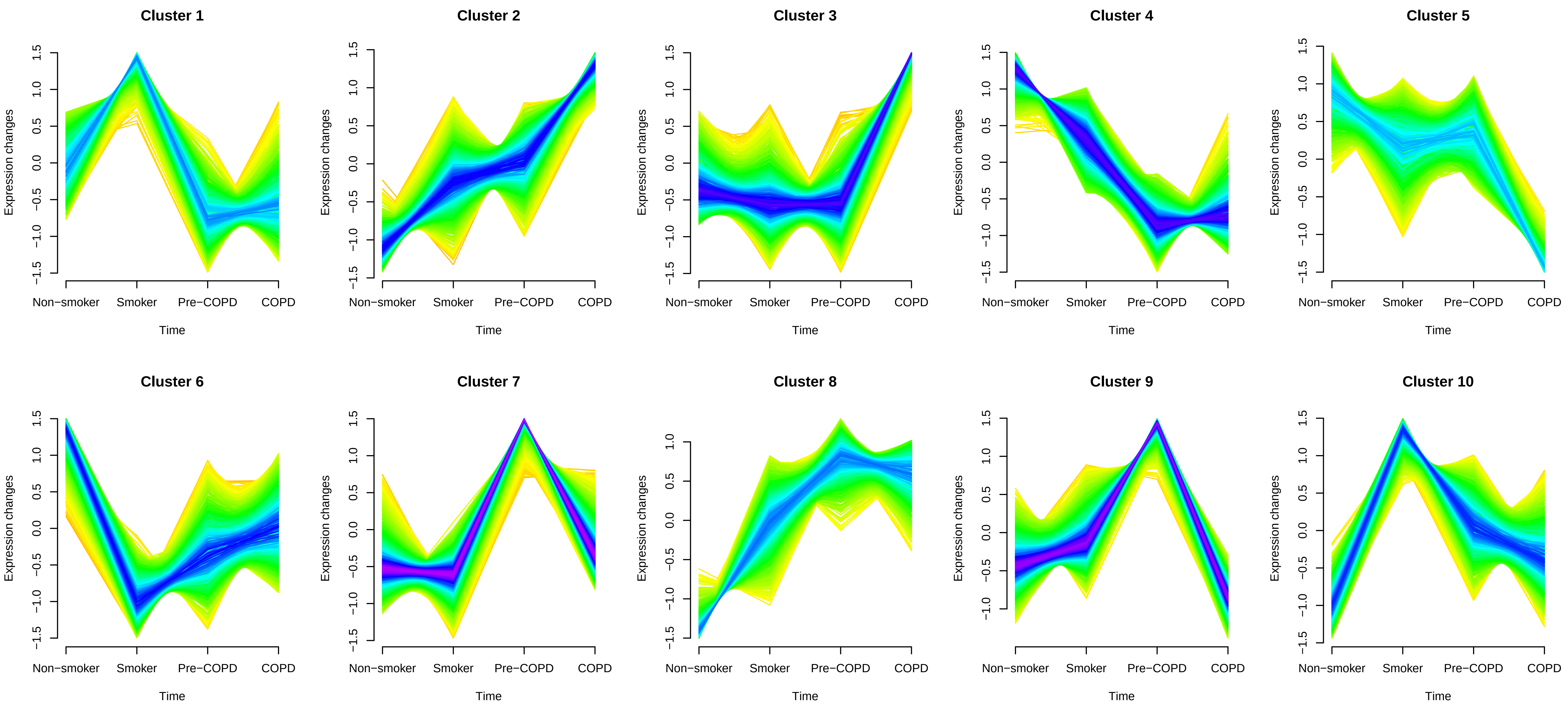

Figure S1. Total of 10 cluster were generated in this Mfuzz based pseudotime analysis.

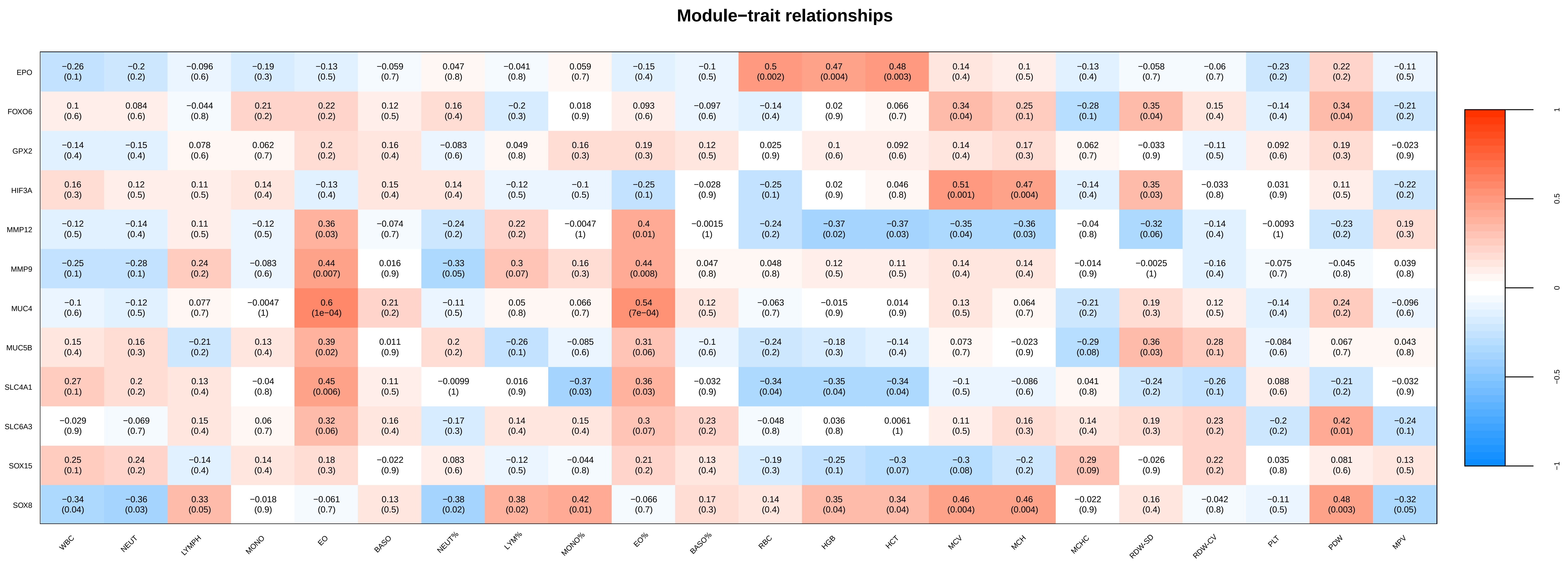

Figure S2. Core gene and complete blood count (CBC) correlation analysis. Sample matched mRNA value for each core gene and CBC results were used for correlation analysis, color in red represent positive correlation, in blue represent negative correlation.

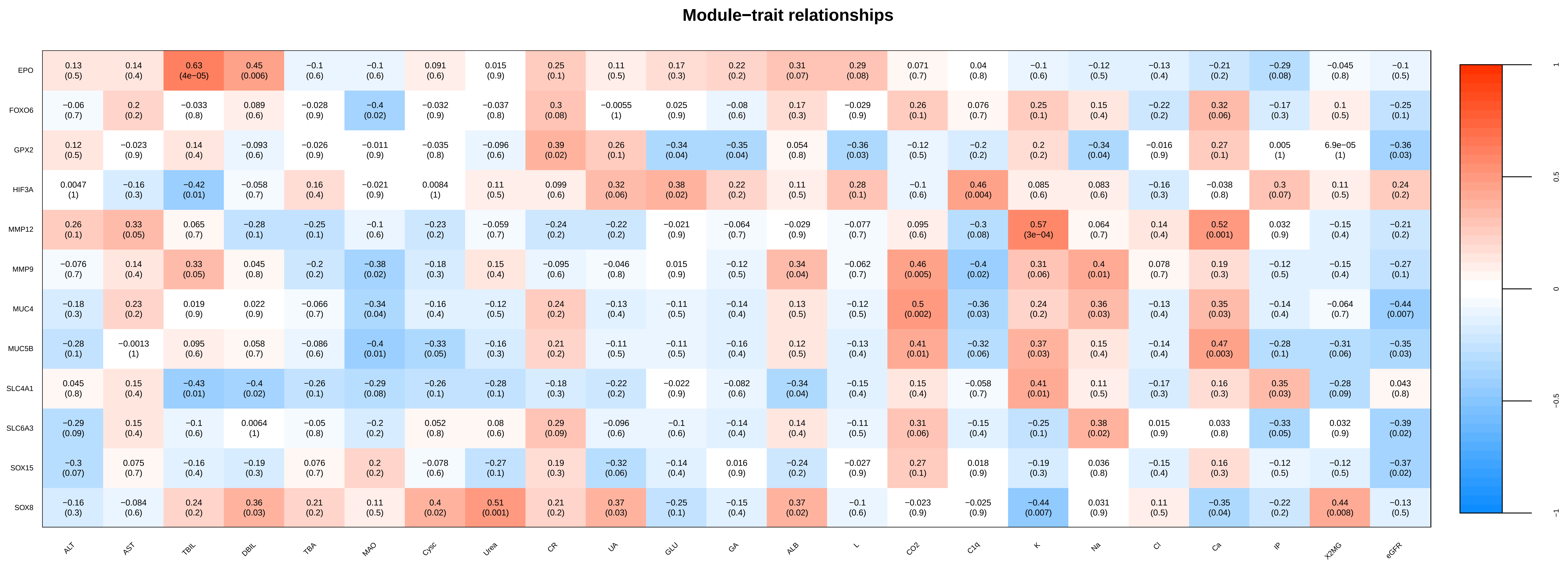

Figure S3. Core gene and blood biochemistry test correlation analysis. Sample matched mRNA value for each core gene and blood biochemistry test results were used for correlation analysis, color in red represent positive correlation, in blue represent negative correlation.

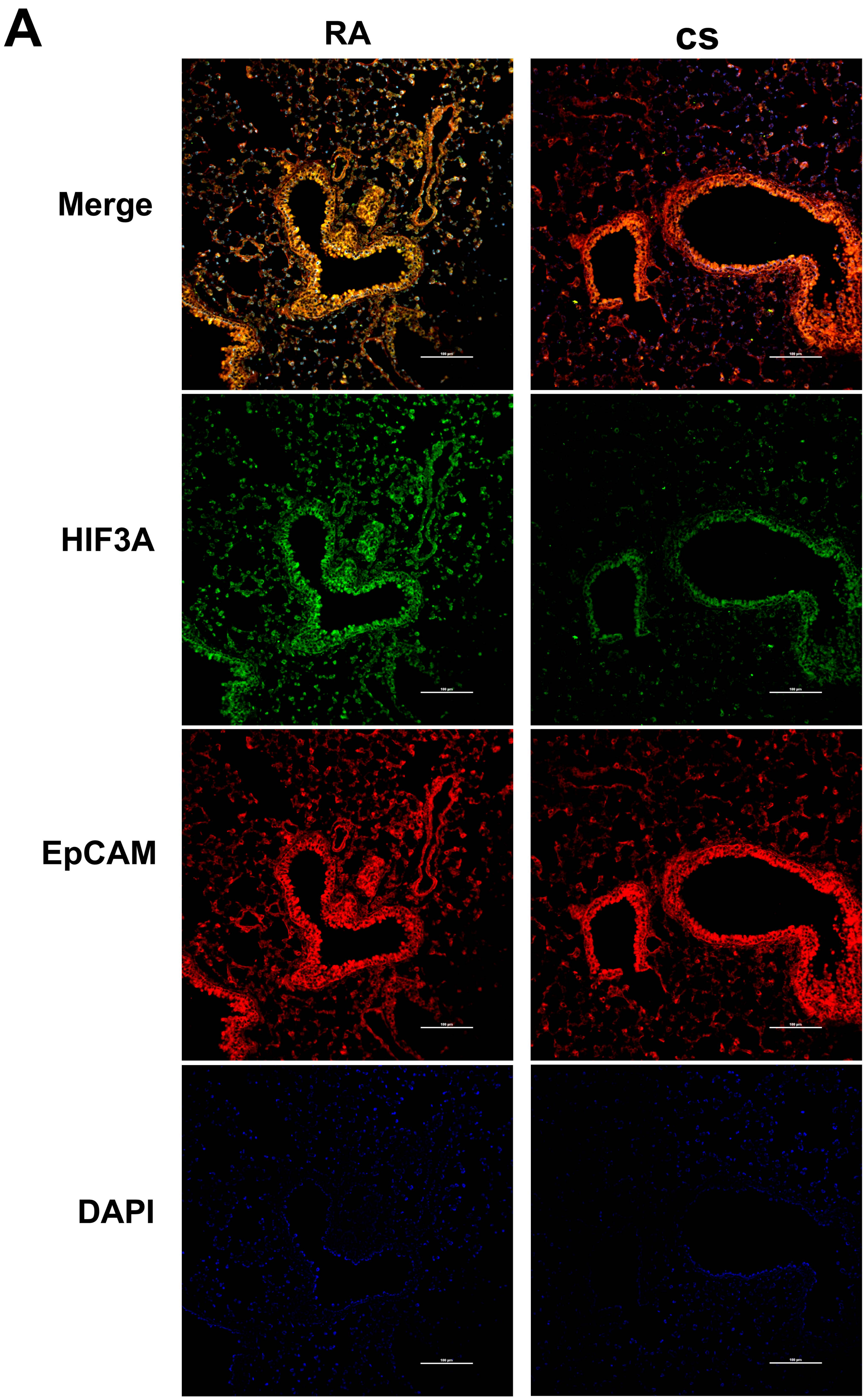

Figure S4. Co-localization analysis of HIF-3α and EpCAM in mice lungs. A) Colocalization of HIF-3α and EpCAM in mouse lung samples; RA stands for room air group; CS stands for cigarette smoking group. Original magnification ×10. Scale bars: 100 μm.

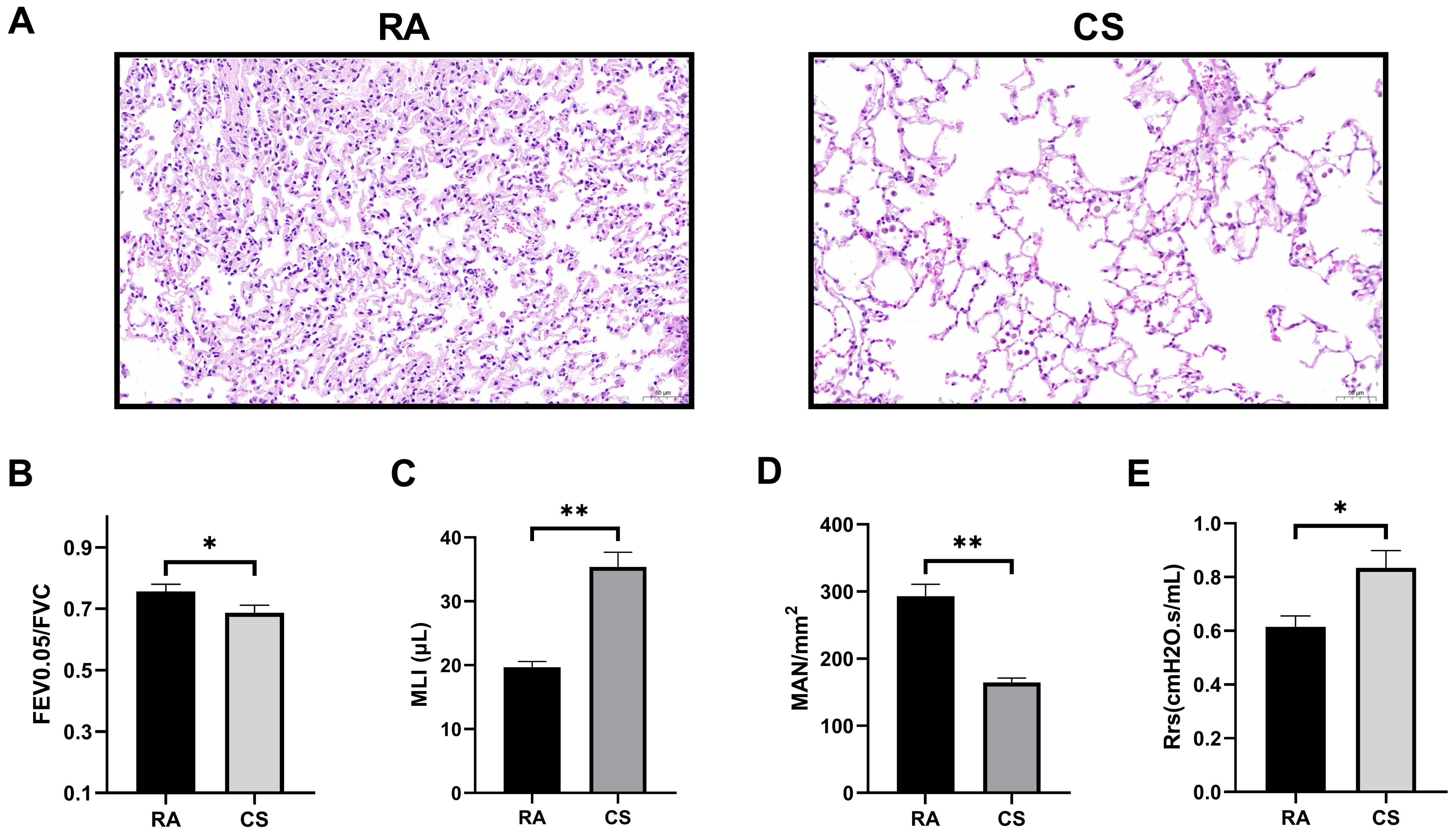

Figure S5. COPD mice model establishment and evaluation. "COPD" mice model was established as described in Method section. **A)** Result of H&E staining in mice model lung specimens were shown, RA stands for room air group, CS stands for cigarette smoking group. We further evaluated the **C)** mean linear intercept (MLI) and **D)** Mean Alveolar Number (MAN) in each group. Using a FlexiVent system, we tested the **B)** $FEV_{0.05}$/FVC, **E)** airway total resistance (Rrs) for each mice at least three times. *: p<0.05; **: p<0.01; ***: p<0.005; ****: p<0.001, ns: p>0.05 (nonsignificant).

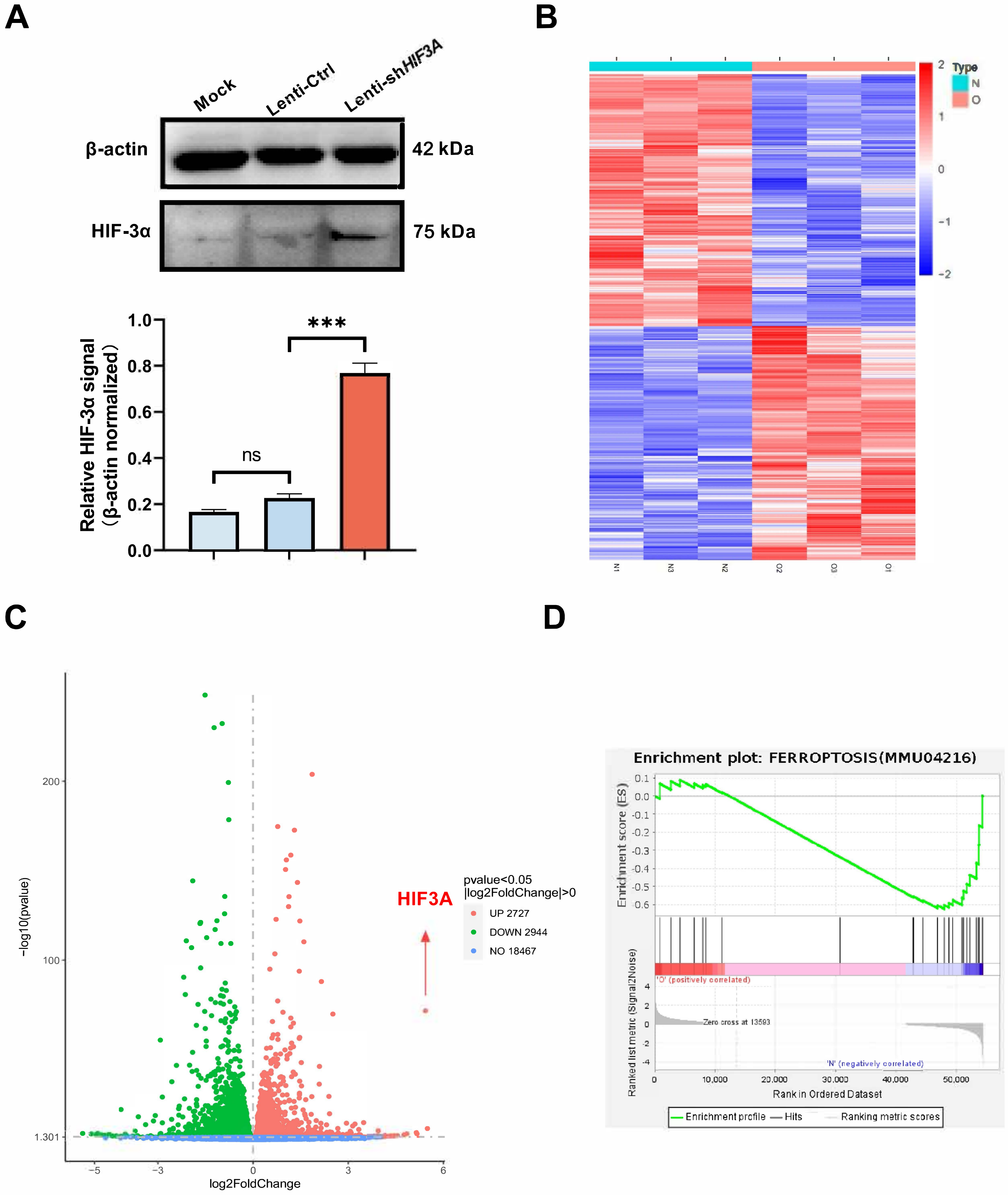

Figure S6. Bulk-RNA sequencing toward HIF-3α overexpression cells shed light on ferroptosis. A) MLE-12 cells were transfected with HIF-3α overexpression (OE) vector (Lenti-shHIF3A) or control vector (Lenti-Ctrl). HIF-3α protein levels were detected by Western blot and normalized to β-actin level. **B)** Heatmap for bulk-RNA sequencing, "N" stands for Lenti-ctrl group, "O" stands for Lenti-shHIF3A group. **C)** Volcano plot shows the differentially expressed genes and the log2FC value of HIF-3α. **D)** GSEA enrichment plot of ferroptosis. ***: $p<0.005$; ns: $p>0.05$ (non-significant)

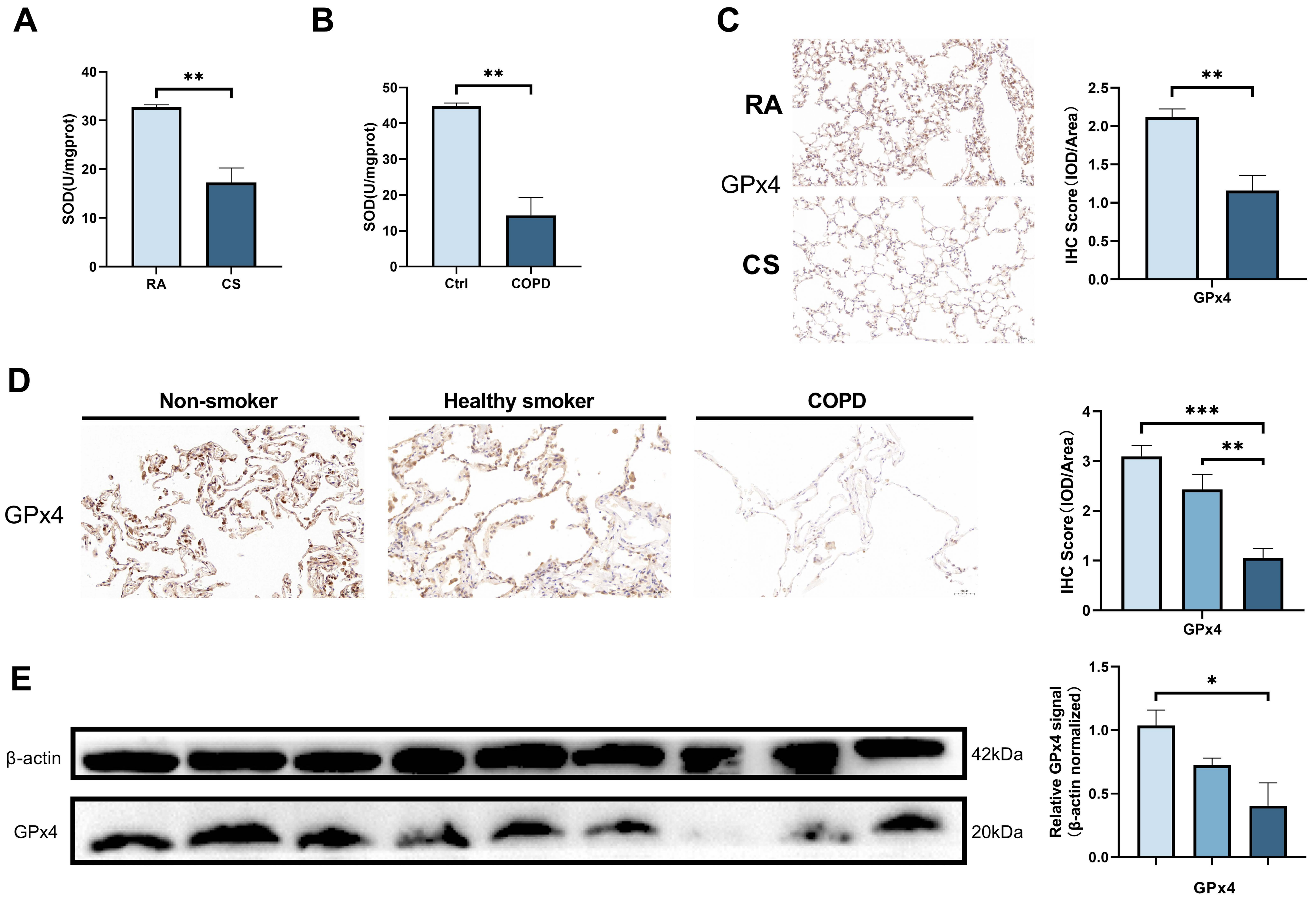

Figure S7. Disrupted GPx4 in COPD samples. Superoxide dismutase (SOD) levels in mice models **A)** humans **B)**. **C)** Immunohistochemistry showed that GPX4 was markedly lower in COPD mice models and in **D)** COPD patients. Original magnification ×20. Scale bars: 50 μm. **E)** Western blotting of human lung homogenates from COPD, non-smokers and smokers were probed with an anti-HIF-3α antibody (normalized to β-actin). IOD: integrated optical density. *: $p<0.05$; **: $p<0.01$; ***: $p<0.005$; ****: $p<0.001$. ns: $p>0.05$ (nonsignificant).

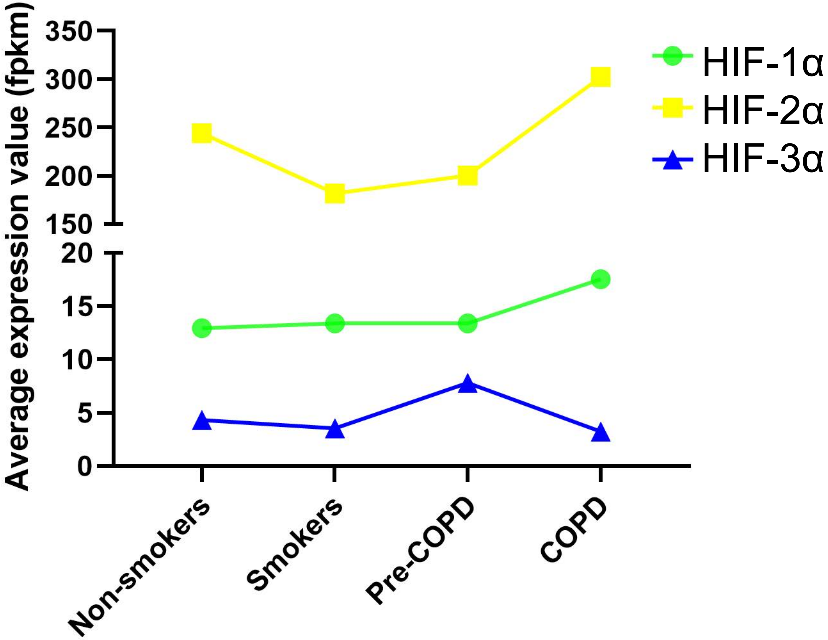

Figure S8. HIFs family expression in four groups: HIF-1α, HIF-2α and HIF-3α average expression value in four groups.

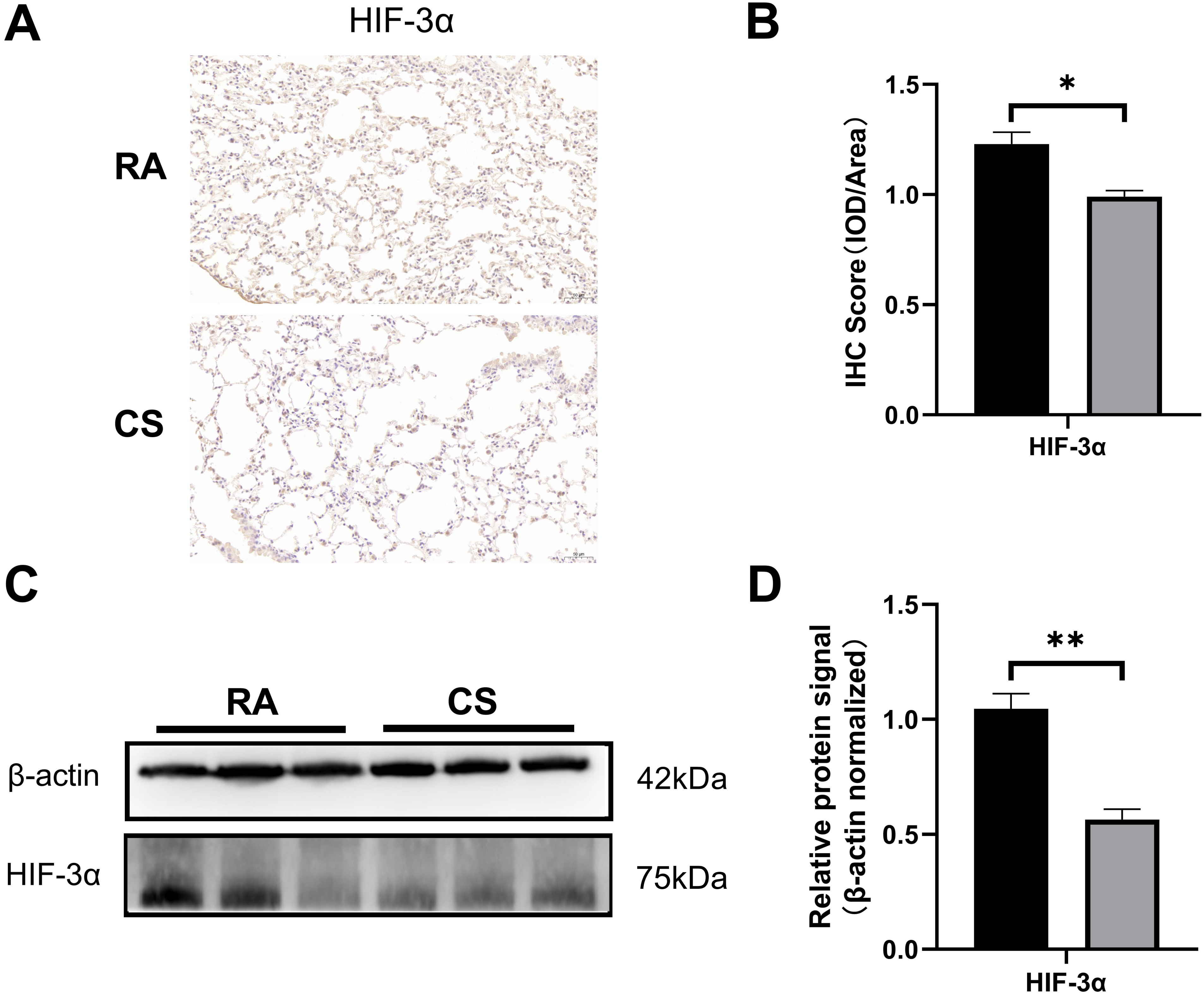

Figure S9. HIF-3α was downregulated in COPD mouse lung. A-B) Immunohistochemistry showed that HIF-3α was significantly lower in COPD mice group than RA group. Original magnification ×20. Scale bars: 50 μm. **C-D)** Western blotting of mice lung homogenates from COPD and RA groups that were probed with an anti-HIF-3α antibody (normalized to β-actin). *: $p<0.05$; **: $p<0.01$.
